# Drug-related and psychopathological symptoms in HIV-positive men who have sex with men who inject drugs during sex (slamsex): Data from the U-SEX GESIDA 9416 Study

**DOI:** 10.1371/journal.pone.0220272

**Published:** 2019-12-04

**Authors:** Helen Dolengevich-Segal, Alicia Gonzalez-Baeza, Jorge Valencia, Eulalia Valencia-Ortega, Alfonso Cabello, Maria Jesus Tellez-Molina, Maria Jesus Perez-Elias, Regino Serrano, Leire Perez-Latorre, Luz Martin-Carbonero, Sari Arponen, Jose Sanz-Moreno, Sara De la Fuente, Otilia Bisbal, Ignacio Santos, Jose Luis Casado, Jesus Troya, Miguel Cervero-Jimenez, Sara Nistal, Guillermo Cuevas, Javier Correas-Lauffer, Marta Torrens, Pablo Ryan, on Behalf of the U-SEX GESIDA 9416 Study

**Affiliations:** 1 Psychiatry Service, Henares University Hospital, Madrid, Spain; 2 Faculty of Medicine, Universidad Francisco de Vitoria, Madrid, Spain; 3 HIV Unit, La Paz University Hospital, IdiPAZ, Madrid, Spain; 4 Harm Reduction Unit, SERMAS, Madrid, Spain; 5 Infectious Diseases and HIV Unit, Fundación Jimenez Díaz, Madrid, Spain; 6 HIV Unit, Clínico San Carlos University Hospital, Madrid, Spain; 7 Infectious Diseases, Ramón y Cajal University Hospital, Madrid, Spain; 8 HIV Unit, Henares University Hospital, Madrid, Spain; 9 HIV Unit, Gregorio Marañón University Hospital, Madrid, Spain; 10 Internal Medicine Service, Torrejon Hospital, Madrid, Spain; 11 Internal Medicine, Príncipe de Asturias Hospital, Alcala de Henares, Spain; 12 Internal Medicine Service, Puerta de Hierro Hospital, Madrid, Spain; 13 HIV Unit, 12 de Octubre University Hospital, Madrid, Spain; 14 Infectious Diseases Unit, La Princesa University Hospital, Madrid, Spain; 15 Internal Medicine Service, Infanta Leonor Hospital, Madrid, Spain; 16 Internal Medicine Service, Severo Ochoa Hospital, Madrid, Spain; 17 Internal Medicine Service, Mostoles University Hospital, Madrid, Spain; 18 Institut de Neuropsiquiatria i Addiccions, Hospital del Mar, Barcelona, Spain; 19 School of Medicine, Complutense University, Madrid, Spain; 20 Instituto de Investigación Sanitaria Gregorio Marañón (IiSGM), Madrid, Spain; Ariel University, ISRAEL

## Abstract

**Objectives:**

Sexualized intravenous drug use, also known as slamsex, seems to be increasing among HIV-positive men who have sex with men (MSM). Physical and psychopathological symptoms have previously been reported in this population, although research on the subject of slamsex is scarce. The objectives of our study were to describe the psychopathological background of a sample of HIV-positive MSM who engaged in slamsex during the previous year and to compare physical, psychopathological, and drug-related symptoms between these participants and those who engaged in non-injecting sexualized drug use.

**Design and methods:**

Participants (HIV-positive MSM) were recruited from the U-Sex study in 22 HIV clinics in Madrid during 2016–17. All participants completed an anonymous cross-sectional online survey on sexual behavior and recreational drug use. When participants met the inclusion criteria, physicians offered them the opportunity to participate and gave them a card with a unique code and a link to access the online survey. The present analysis is based on HIV-positive MSM who had engaged in slamsex and non-injecting sexualized drug use.

**Results:**

The survey sample comprised 742 participants. Of all the participants who completed the survey, 216 (29.1%) had engaged in chemsex, and of these, 34 (15.7%) had engaged in slamsex. Participants who engaged in slamsex were more likely to have current psychopathology (depression, anxiety, and drug-related disorders) than participants who engaged in non-injecting sexualized drug use. In addition, participants who engaged in slamsex more frequently reported high-risk sexual behaviors and polydrug use and were more often diagnosed with sexually transmitted infections (STIs) and hepatitis C than those who did not inject drugs. Compared with participants who did not inject drugs, participants who engaged in slamsex experienced more severe drug-related symptoms (withdrawal and dependence), symptoms of severe intoxication (loss of consciousness), and severe psychopathological symptoms during or after slamsex (eg, paranoid thoughts and suicidal behaviors).

**Conclusion:**

Slamsex is closely associated with current psychiatric disorders and severe drug-related and psychiatric symptoms.

## Introduction

Chemsex, or sexualized drug use, was first described in the UK as the intentional use of recreational drugs to enhance sexual relations between men who have sex with men (MSM), usually for long periods of time and often with multiple partners [1]. The main drugs involved in this practice are mephedrone, γ-hydroxybutyrate/γ-butyrolactone (GHB/GBL), and crystal-methamphetamine (crystal-meth) [2], although other drugs have also been reported, including ketamine, other synthetic cathinones, 3,4-methylenedioxymethamphetamine (MDMA), cocaine, poppers, and erectile-dysfunction drugs, [3]. Other aspects of this phenomenon, such as the use of geosocial networking applications to locate or participate in sex parties, should be taken into consideration because of their relevance and implications [4]. The use of smartphone applications (apps) designed to enable MSM to find casual sexual partners has been linked to a higher number of sexual partners, a higher probability of engaging in unprotected anal intercourse, and a higher probability of having been diagnosed with a sexually transmitted infection (STI) [5].

Intravenous use of psychoactive substances in this context, especially stimulants such as mephedrone and crystal-meth, is known as slamming or slamsex [2]. Some studies have suggested that the practice of injecting recreational drugs at sex parties might be increasing among MSM. For instance, Glass et al. [6] reported that since 2000, the proportion of MSM who inject drugs has increased (from 4.4% in 2000/01 to 8.4% in 2014/15, *P*<0.001) among users of general drug services in England. Both slamsex and non-injecting sexualized drug use are more prevalent in MSM living with human immunodeficiency virus infection (HIV-positive) than HIV-negative MSM [7].

Slamsex has been associated with group sex, condomless sex with casual partners, and fisting, all of which increase the frequency of STIs and the transmission of viral infections, such as those caused by HIV and hepatitis C virus (HCV) [7].

Both mephedrone and crystal-meth are potent central nervous system stimulants that also act peripherally. The potency and half-life of mephedrone depends on the route of administration. If administered orally, onset of action takes half an hour, with a mild high that can last from 3 to 5 hours. Intranasal administration leads to a potent high after 15 minutes that lasts 1–2 hours. With intravenous administration, the high is almost immediate and very potent and lasts 30 to 45 minutes. The rapid onset of action and fast dissipation of effects leads to a compulsive pattern of use and the need to re-dose almost every hour. Thus, high doses of mephedrone are used in sexual settings, with the consequent risk of overdosing, altered behavior, and delusional thoughts.

Crystal-meth differs from mephedrone in that its potency is similar irrespective of whether it is injected intravenously or smoked. Either route of administration produces immediate onset of action, within 0 to 2 minutes, and leads to a very potent high. If it is injected intravenously, the effect can last almost 8 hours. Crystal-meth produces an intense state of excitement, with euphoria and increased self-confidence and sociability. Given that its withdrawal syndrome is very unpleasant, its addictive potential is very high [8].

Both substances have been related to induced psychotic symptoms in diverse populations [9,10]. However, the appearance of psychiatric symptoms associated with slamsex is scarce, although there is evidence suggesting that mephedrone used in slamsex can induce psychotic symptoms and suicidal behavior [11]. Crystal-meth has also been related to high levels of addiction, psychotic symptoms, and other psychiatric disorders in the context of chemsex [12].

Mental health issues have been poorly studied among persons who engage in chemsex, and few data are available on the severity of drug-related symptoms in HIV-positive MSM who engage in slamsex.

The aim of our study was to compare the physical and psychopathological background (history of STIs, sexual behaviors, and diagnosed mental disorders such as depression and anxiety) of HIV-positive MSM who engaged in slamsex with that of those who engaged in non-injecting sexualized drug use. We also explored the presence of psychopathological symptoms and symptoms of substance use disorders during or after drug use in the study population and investigated associated factors. Patients were selected from the U-SEX GESIDA study [13].

## Materials and methods

The present analysis is nested in the U-SEX GESIDA 9416 study, which was conducted in 22 HIV clinics in the Madrid area from June 2016 to March 2017. The study aimed to calculate the prevalence of chemsex and associated factors in a sample of HIV-positive MSM in Spain. The inclusion criteria were age ≥18 years, documented HIV infection, and being an MSM (homosexual or bisexual). Infectious diseases physicians offered all the participants who met the inclusion criteria the opportunity to participate and gave them a card with a unique ID code and a link that enabled access to an online survey. Cards were non-transferable. The unique ID code was given to each participant to allow them access to the website and complete the survey. The ID code prevented people not selected for the study from responding to the survey and was not linked to the participant’s identifying data. The physician evaluated the rate of response and the representativeness of the sample (code, age, year of HIV diagnosis, level of education, and nationality). The survey was self-completed outside the hospital to ensure anonymity and confidentiality.

The online survey was designed ad hoc by the research team to evaluate various domains: general sociodemographic data (age, occupational status, income), HIV infection status (year of diagnosis, treatment, adherence), sexual behaviors (condom use, receptive anal sex, fisting), diagnosis of STIs (including HCV), diagnosed psychiatric disorders, and history of drug use. If the participant reported any kind of drug use, he was asked if these drugs were used before or during sexual encounters. Chemsex was defined as the intentional use of mephedrone or other cathinones, MDMA, methamphetamine, GHB/GBL, ketamine, or cocaine during sex. This analysis included participants who reported having engaged in chemsex in the previous 12 months. The survey evaluated the type of drugs used, the context in which they were used, frequency of use, route of administration, and other aspects related to chemsex (eg, setting).

In order to collect data on psychiatric disorders, the survey asked general questions regarding previously diagnosed psychiatric disorders and specific questions about “past” or “current” psychiatric disorders diagnosed by a mental health specialist. We only considered self-reported current psychiatric disorders (diagnosed in the previous year), namely, depression, anxiety, personality disorders, psychosis, and drug-related disorders. Because the survey was self-completed, we used the term “self-reported current psychiatric disorder”.

All participants were asked about dependence, withdrawal, and psychopathological symptoms related to the drugs used during chemsex. To determine symptoms of drug dependence, the survey asked about the following items: drugs used more often or in a higher quantity than planned, severe craving, not fulfilling obligations because of drug use, continuing drug use (even when this leads to physical or psychological discomfort), need to increase doses to obtain the same effect, and less positive effects with the same doses. For purposes of the analysis, dependence was defined as the presence of three or more symptoms of drug dependence during the previous year.

In order to collect data on withdrawal symptoms we asked about the following: severe craving, need to take medications/other drugs to compensate for discomfort, sleep disturbances (insomnia, hypersomnia), agitation, depressive thoughts/feelings, paranoid ideation, suicidal thoughts, suicide attempts, and the need to see a doctor for treatment of discomfort. The presence of three or more withdrawal symptoms during the previous year was also recorded.

Finally, intoxication-related symptoms were assessed based on the following: sleep disturbances, “things done to me that I would not have consented to without being on drugs”, “more sexual risk practices that I don’t do when not on drugs”, unpleasant physical feelings under the effects of drugs, anxiety/panic attacks, irritability, and aggressiveness. Psychotic symptoms (mainly paranoid ideation), loss of consciousness, suicidal thoughts, and suicide attempts were considered to be symptoms of severe intoxication.

Details of the study procedures have been published elsewhere [13]. In order to clarify terminology, participants were considered to have engaged in slamsex when they engaged in sexualized intravenous drug use and in non-injecting sexualized drug use when the drugs were not consumed intravenously.

The study protocol was approved by the Ethics Committee of Hospital Universitario Gregorio Marañón (HUIL 1606 96/16) and fulfilled the principles of the Declaration of Helsinki (2008).

Study data were collected and managed using the data capture tool Research Electronic Data Capture (REDCap) [14], which is hosted at “Asociación Ideas for Health”.

### Statistical analysis

Categorical variables were expressed as absolute and relative frequencies; continuous variables were expressed as median (IQR). Baseline characteristics were compared between participants who had engaged in slamsex and participants who had engaged in non-injecting sexualized drug use during the previous year using the chi-square test for categorical variables and the *t* test for continuous variables. Variables included in the comparisons were sociodemographic variables, self-reported current psychiatric disorders, physical and severe psychopathological symptoms related to drug use/abuse, sexual behaviors, and medical variables such as time since HIV diagnosis, self-reported adherence to antiretroviral therapy, and diagnosis of an STI.

We conducted a logistic regression analysis to explore the association between slamsex and symptoms of drug use disorders/severe psychopathological symptoms. We performed separate tests to examine the association between slamsex and the following: presence of withdrawal symptoms (three or more withdrawal symptoms), dependence (three or more dependence-related symptoms), craving (increased need for consumption), paranoid ideation (during or after drug use), suicidal behaviors (suicidal ideation and suicide attempts during or after drug use), and loss of consciousness (during or after drug use).

The univariate analysis was conducted separately to evaluate the association between symptoms of drug-related disorders or severe psychopathological symptoms in the context of chemsex and other drug-related variables or self-reported current psychiatric disorders. The dependent variables included withdrawal symptoms, severe craving, psychotic paranoid ideation, suicidal behaviors, and loss of consciousness. Independent variables were categorized as the presence/absence of self-reported active depression, self-reported active anxiety, polydrug use (three or more drugs used during a session), cathinone use during the previous year, ketamine use during the previous year, GHB use during the previous year, and smoking crystal-meth during the previous year. Thereafter, bivariate logistic regression analysis was conducted to explore associations regardless of the presence of slamsex. The presence/absence of slamsex was included in the bivariate regression as an independent variable. Independent variables were included in the bivariate analysis only if their p value was ≤.10 in the univariate analysis.

## Results

### 1.1. Baseline characteristics and comparison between participants who engaged in slamsex and participants who engaged in non-injecting sexualized drug use

Of a total of 742 HIV-positive MSM who completed valid surveys in the U-Sex Study, the present analysis included all the participants who had engaged in chemsex during the previous year (N = 216). Participants in our sample were mainly Spanish-born (71.3%), middle aged (median = 38; IQR: 33–44), and educated to university level (63.9%). In addition, 70.8% had a salary of more than 1000 euros per month, and 42% were in a stable relationship. The median time with HIV diagnosis was five years (IQR: 2–11). More than 90% were receiving antiretroviral therapy, and of these, 3% reported having taken less than 90% of their doses (poor adherence). In our sample, 34 participants (15.7%) had engaged in slamsex during the previous year. A comparison with HIV-positive MSM who did not engage in chemsex in our sample has been reported elsewhere [13].

When participants who had engaged in slamsex during the previous year were compared with those who engaged in non-injecting sexualized drug use, no differences were found regarding sociodemographic or medical variables. Compared with people who engaged in non-injecting sexualized drug use, people who had engaged in slamsex were less likely to have a stable partner (26.5 vs. 45.6%, *P* = .039) and were more likely to have suboptimal adherence to antiretroviral therapy (9.1 vs. 1.9%, *P* = .061).

Comparisons based on the type of drug used in both groups of participants are shown in Table 1.

**Table 1:** Comparisons between participants who engaged in non-injecting sexualized drug use and participants who engaged in slamsex in terms of type of drug used in the previous year.

	Total sample (N = 216)	Non-injecting sexualized drug use (n = 182)	Slamsex (n = 34)	P value
**Polydrug, No. (%)**	98 (45.4)	70 (38.5)	28 (82.4)	.000
**Poppers, No. (%)**	170 (78.7)	140 (76.9)	30 (88.2)	.139
**Mephedrone or other cathinones, No. (%)**	150 (69.4)	116 (63.7)	34 (100)	.000
**Cocaine, No. (%)**	171 (79.1)	146 (80.2)	25 (73.5)	.378
**MDMA, No. (%)**	105 (48.6)	87 (47.8)	18 (52.9)	.582
**GHB, No. (%)**	155 (71.7)	128 (70.3)	27 (79.4)	.280
**Crystal-methamphetamine,** **No. (%)**	64 (29.6)	47 (25.8)	17 (50)	.005
**Ketamine, No. (%)**	78 (36.1)	57 (31.3)	21 (61.8)	.001
**Rectal use of drugs, No. (%)**	44 (20.4)	24 (13.2)	20 (58.8)	.000
**High-risk drug use, No. (%)**	168 (77.8)	135 (74.2)	33 (97.1)	.003

Participants who engaged in slamsex had higher rates of polydrug use (three or more drugs per session) and more frequently used mephedrone and other cathinones, crystal-meth, and ketamine. They were also more likely to administer the drugs rectally and had higher rates of high-risk drug use behaviors, such as sharing needles and other drug paraphernalia. Symptoms related to drug abuse/dependence and severe psychopathological symptoms associated with the practice of slamsex and non-injecting sexualized drug use are shown in Table 2.

**Table 2:** Self-reported psychiatric symptoms during and after non-injecting sexualized drug use and slamsex.

	Total sample (N = 216)	Non-injecting sexualized drug use (n = 182)	Slamsex (n = 34)	P value
**3 or more dependence symptoms, No. (%)**	60 (27.8)	40 (22)	20 (58.8)	.000
**3 or more withdrawal symptoms, No. (%)**	98 (45.8)	72 (39.6)	26 (76.5)	.000
**Intense craving, No. (%)**	55 (25.5)	34 (18.5)	21 (61.8)	.000
**Interference with work, social, or family life, No. (%)**	68 (31.5)	46 (25.3)	22 (64.7)	.000
**Paranoid ideation, No. (%)**	30 (15.3)	20 (11)	10 (29.4)	.004
**Suicidal ideation, No. (%)**	33 (15.3)	22 (12.1)	11 (32.4)	.003
**Suicide attempt, No. (%)**	30 (13.8)	19 (10.4)	11 (32.4)	.001
**Loss of consciousness, No. (%)**	33 (15.3)	23 (12.6)	10 (29.4)	.013

The drugs most frequently injected intravenously were mephedrone and other cathinones (94.1%), followed by ketamine (17.6%), crystal-meth (5.9%), and cocaine (5.9%).

Participants who engaged in slamsex had a significantly higher percentage of sexual risk behaviors than those who engaged in non-injecting sexualized drug use, as follows: fisting (73.5 vs. 38.5%, *P* = .001), fisting without a glove (67.7 vs. 28%, *P* = .001), condomless sexual relations (93.1 vs. 48.3%, *P* = .001), and more than 20 sexual partners in the previous 6 months (70 vs. 39.6%, *P* = .002). As for STIs, people who had engaged in slamsex more often had gonorrhea (61.8 vs. 43.4%, *P* = .049), syphilis (88.2vs. 62.6%, *P* = .004), and hepatitis C (61.8% vs. 18.1%, *P* = .000) than people who engaged in non-injecting sexualized drug use.

A self-reported current psychiatric disorder was more common among participants who engaged in slamsex than in those who engaged in non-injecting sexualized drug use. The conditions reported were as follows: depressive disorder (61.8 vs. 28%, *P* = .0001), anxiety disorder (47.1 vs. 23.1%, *P* = .004), and drug use disorders (drug-dependence) (38.2 vs. 15.4%, *P* = .002).

### 1.2. Correlates of severe physical and psychopathological symptoms related to drug use

The simple logistic regression conducted to explore the association between slamsex and the presence of symptoms of drug use disorders or severe psychopathological symptoms related to drug use revealed a significant association. Compared with participants who had engaged in non-injecting sexualized drug use, those who engaged in slamsex were five times more likely to have experienced withdrawal symptoms (OR: 4.97 [2.13–11.57], *P* = .0001) and seven times more likely to have experienced intense craving (OR: 7.03 [3.21–15.43], *P* = .0001). Moreover, during or after drug use they were three times more likely to experience suicidal ideation (OR: 3.48 [1.48–8.10], *P* = .004), psychotic paranoid ideation (OR: 3.38 [1.41–8.07], *P* = .006), and loss of consciousness (OR: 2.88 [1.22–6.79], *P* = .016).

**Fig 1:**
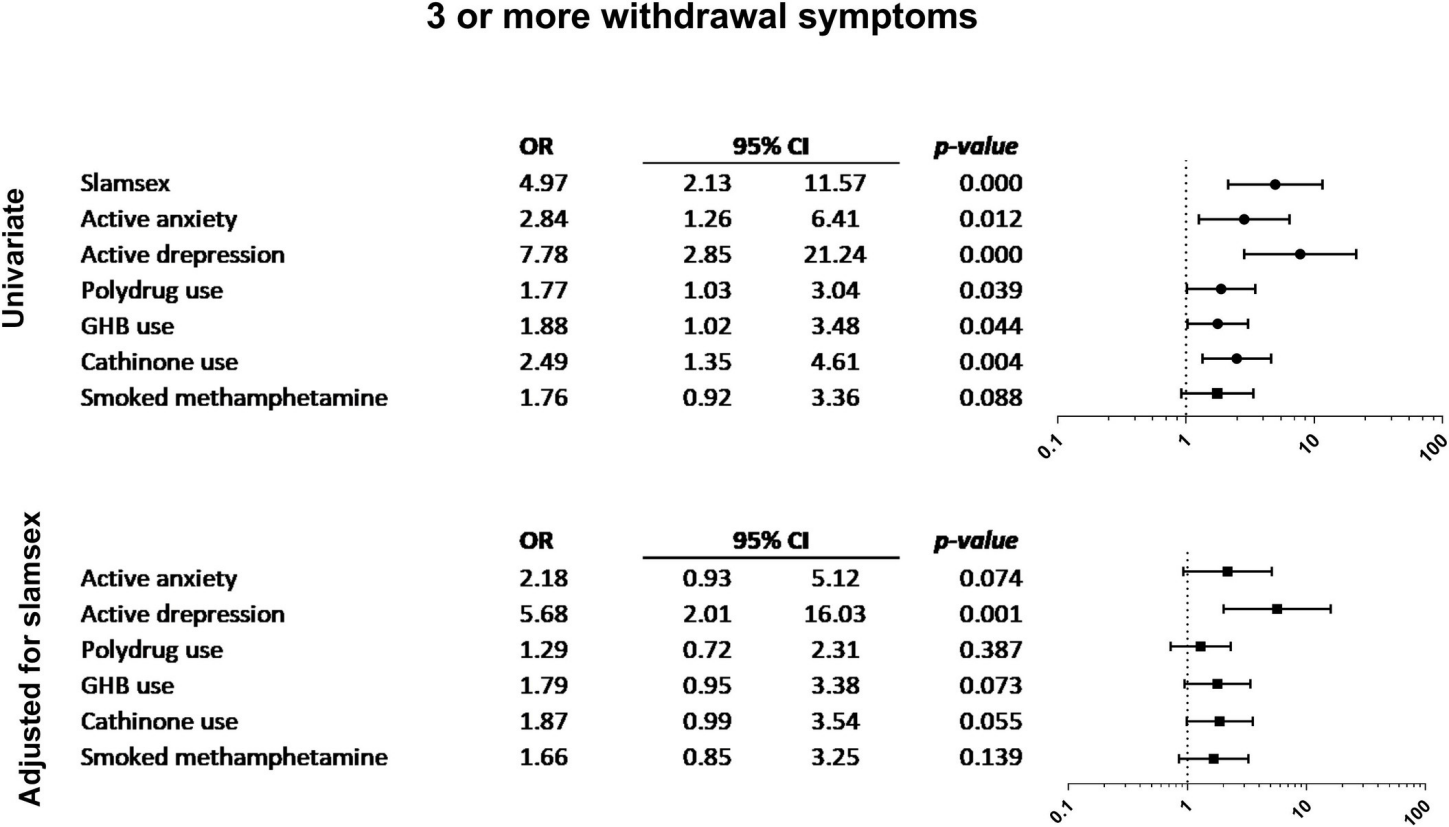
Association between current self-reported psychiatric diagnosis and 3 or more withdrawal symptoms.

**Fig 2:**
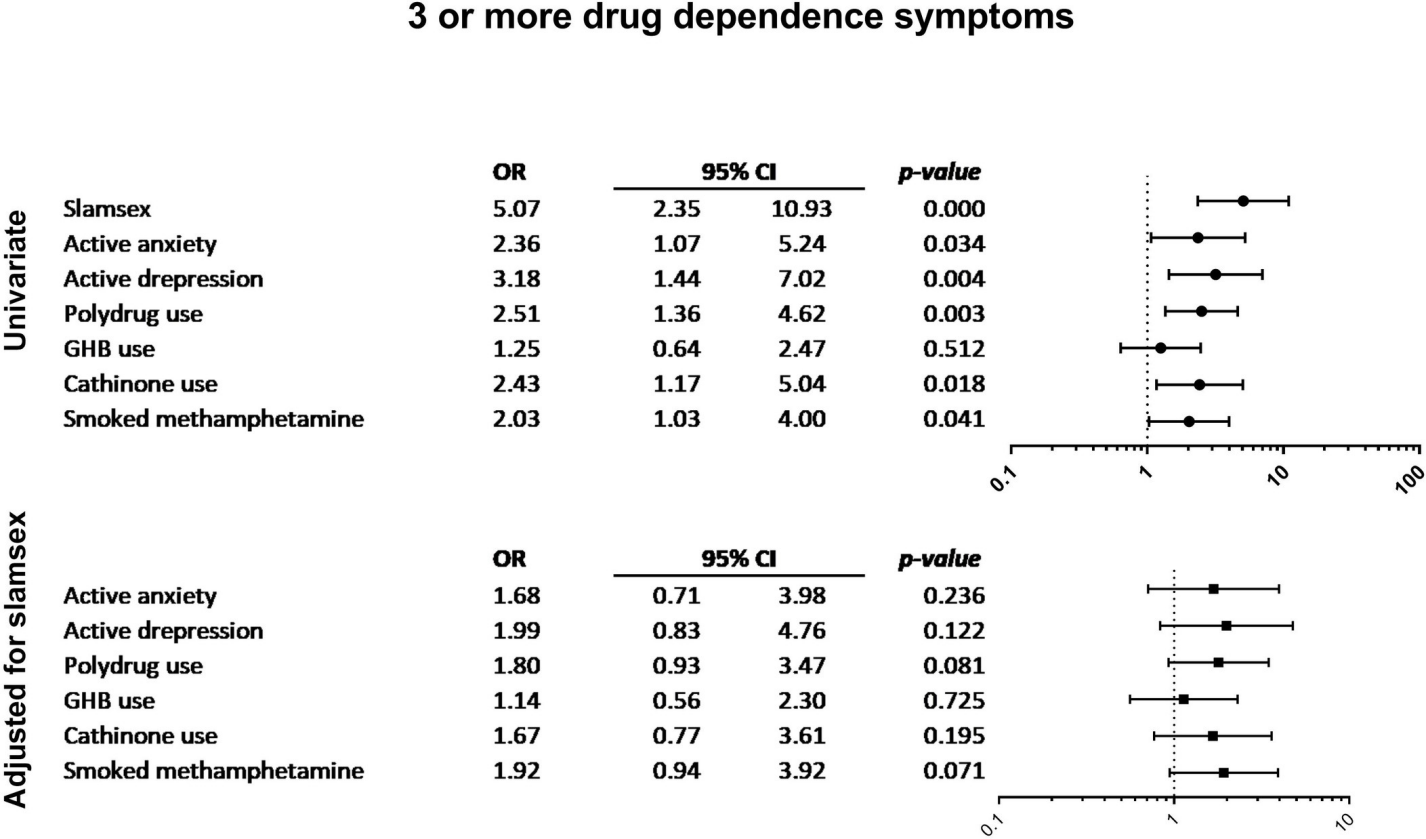
Association between current self-reported psychiatric diagnosis and 3 or more drug-dependence symptoms.

**Fig 3:**
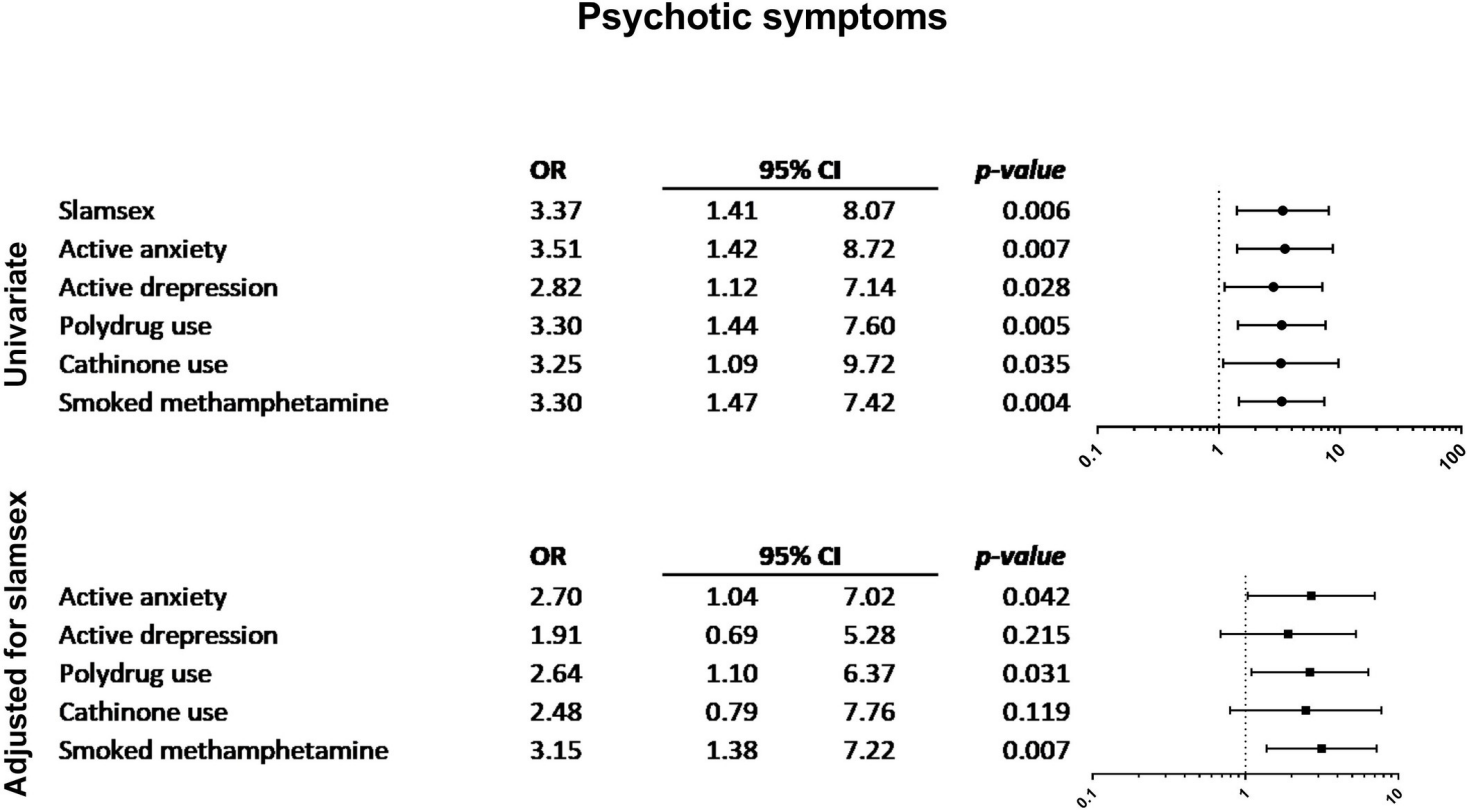
Association between current self-reported psychiatric diagnosis and psychotic symptoms.

**Fig 4:**
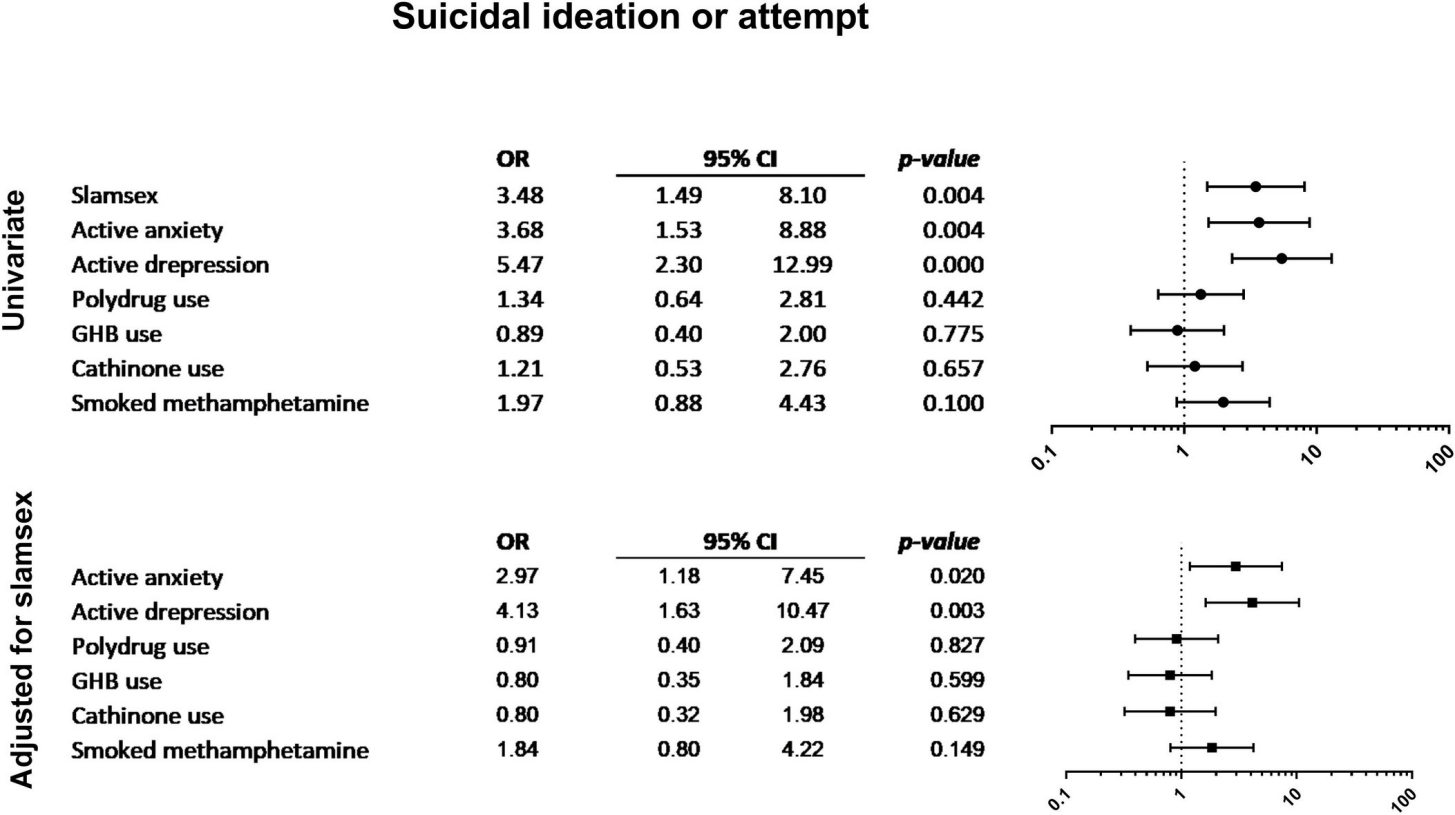
Association between current self-reported psychiatric diagnosis and suicidal ideation or attempt.

Figs 1–4 show the associations between other drug-related variables/current self-reported psychiatric diagnosis and the presence of symptoms of drug-related disorders and severe physical and psychopathological symptoms related to drug use (suicidal ideation, paranoid ideation, 3 or more withdrawal symptoms, and 3 or more drug-dependence symptoms), regardless of the presence of slamsex.

Participants who self-reported current depressive disorders more frequently had withdrawal symptoms. Active anxiety, cathinone use, and GHB use were also associated with the presence of withdrawal symptoms. Moreover, participants who smoked crystal-meth more frequently experienced severe craving, and those who smoked crystal-meth or used multiple drugs were significantly more likely to present symptoms of drug dependence. Suicidal ideation was only associated with self-reported depression and anxiety disorders; paranoid ideation was associated with anxiety disorders, polydrug use, and smoked crystal-meth. Finally, loss of consciousness was related to polydrug use, GHB use, ketamine use, and smoked crystal-meth.

## Discussion

The present study provides novel findings regarding the slamsex phenomenon in a sample of HIV-infected MSM who engage in sexualized drug use. In our sample, 216 subjects engaged in chemsex. From this sub-sample, 34 subjects (15.7%) had engaged in slamsex during the previous year. Compared with those who did not inject drugs, people who had engaged in slamsex more frequently reported high-risk sexual behaviors, had more frequently been diagnosed with an STI, and had more frequently reported a current diagnosis of a psychiatric disorder. In addition, compared with participants who engaged in non-injecting sexualized drug use, participants who had engaged in slamsex in the previous year had more drug-related adverse effects such as symptoms of withdrawal and dependence or severe physical and psychopathological symptoms, such as psychotic paranoid ideation, suicidal behaviors, and loss of consciousness. Based on the route of consumption, we compared these two sub-groups to evaluate potential differences between people who used the same drugs in a sexual context and to analyze whether there were differences regarding psychopathological background. We also determined whether the group who injected drugs had more drug-related symptoms, as might be expected. Because of the design of the survey and the fact that psychiatric conditions were self-reported, we cannot suggest causality and can only report whether there were differences between the groups and whether psychiatric background correlated with drug-related symptoms. This limitation raises the need for structured studies among MSM who attend HIV clinics. Such studies should be performed by trained mental health professionals operating within multidisciplinary programs who can assess mental health through personal interviews and structured scales. In this way, we would be able to diagnose and treat possible mental disorders in this population. The assessment of drug-related disorders can be addressed in the same manner, thus making it possible to detect drug-related problems early, apply harm-reduction approaches, and prevent potential problems related to sexualized drug use.

Data have been published on the prevalence of slamsex and associated high-risk behaviors among MSM. The Unlinked and Anonymous Monitoring (UAM) survey of people who inject drugs conducted in general drug services across England, Wales, and Northern Ireland reported a significant increase in the proportion of MSM who inject drugs from the year 2000 to 2015. They also reported the presence of high-risk behaviors associated with injecting such as needle/syringe sharing (15% vs. 11%, *P* = 0.07) and having more than 10 sexual partners among MSM who injected drugs than among MSM who did not inject drugs (25% vs. 4.0%, *P*<0.001) [6]. A recent study from an Australian cohort of MSM reported a high life prevalence of injecting drugs (10.3%); the prevalence of injection in the previous six months was 4.7% in this population. The authors reported that injecting drugs was associated with high-risk sexual practices such as having multiple sex partners, group sex with casual partners, and condomless anal intercourse with casual partners [15]. In the case of HIV-positive MSM, the ASTRA study [16] reported that of 2248 HIV-positive sexually active MSM recruited in 2011–2012, 1138 (51%) had used recreational drugs in the previous three months and that the prevalence of injection drug use was 3% (n = 68). The Positive Voices study reported that 105 of 392 sexually active HIV-positive MSM (29%) had engaged in chemsex during the previous year. Among these, the prevalence of slamsex was 33.3% [17]. The prevalence of slamsex in our study could be directly compared with the findings of the Positive Voices study only because of methodological and sample-related similarities. While the frequency of chemsex reported is similar to that we reported (29%) in the U-Sex Study [13], the authors found higher frequencies of slamsex among their participants (33.3 vs. 15.6%). Therefore, we think that regional differences in slamsex frequencies should be explored in future studies. The most dangerous profiles of drug use and sexual practices found in the abovementioned studies in samples of MSM who injected drugs are congruent with the higher frequencies of polydrug use, rectal administration, sharing drug paraphernalia, sexual risk behaviors, and STIs found among those who engaged in slamsex in the present study.

We found that the most common intravenous drugs used during slamsex were mephedrone and other synthetic cathinones (94.1%), followed by ketamine (17.6%), crystal-meth (5.9%), and cocaine (5.9%). To our knowledge, only a few reports discuss the type of drug used by HIV-positive MSM during slamsex. The UAM Survey found high frequencies of injected mephedrone and ketamine among MSM who injected drugs (12% and 9.3%, respectively) [6]. Furthermore, data from Antidote, a specialist drug clinic aimed at the gay community in London, UK showed that 75% of patients used mephedrone in chemsex and that of these, 80% injected the drug. Of this 80%, 75% were HIV-positive and 70% reported sharing needles [18]. The recently published FLUX study, a survey performed in Australian gay and bisexual men, found that of the 1995 respondents, 206 (10.3%) reported having injected drugs and 93 (4.7%) had injected recently, most commonly crystal-meth (91.4%) and speed (9.7%), as well as cocaine and ketamine, albeit in low percentages [15]. Together with the data reported above, our results suggest that the type of drugs injected in slamsex are similar, but that there may be regional differences. Drug use in the context of slamsex and non-injecting sexualized drug use may be changing as a result of travel by MSM to different countries for leisure, socialization, and clubbing and to expand sexual experiences.

The participants in our sample who engaged in slamsex presented a higher frequency of drug-related adverse effects than those who engaged in non-injecting sexualized drug use. Severe craving and other withdrawal symptoms were more frequent, as was loss of consciousness. The participants also had higher rates of severe psychopathological symptoms such as paranoid ideation and suicidal ideation or attempts.

Mephedrone and other synthetic cathinones were the main drugs “slammed” in our sample, both as stimulants and as sexual enhancers. The intravenous injection of mephedrone has been related to compulsive use, intense craving, binging behaviors, and withdrawal symptoms [19]. Diverse psychotic symptoms, mainly paranoid ideation, have also been reported for mephedrone, especially if it is consumed intravenously [20,21]. In the context of slamming, one case in Spain was reported in a young HIV-positive man who experienced persistent mephedrone-induced paranoid delusions, intense anxiety, and visual and kinesthetic hallucinations [11].

Ketamine, cocaine, and crystal-meth were also consumed in slamsex in our sample, albeit at a lower frequency than cathinones. Injected crystal-meth has the potential to induce psychotic symptoms and has been associated with drug-related disorders such as abuse or dependence. In slamsex, its potent stimulant effect has been related to high-risk sexual behaviors, with an increased risk of HIV infection and other STIs [8].

Traditionally, more frequent drug dependence and psychiatric symptoms have been described during intoxication by or abstinence from some drugs if they are used intravenously. Our novel data, together with the few previously published findings, support the addictive potential and severe psychopathological symptoms associated with sexualized intravenous drug use.

Other variables related to drug use might modulate the severity of physical and psychopathological symptoms in the context of non-injecting sexualized drug use. In our sample of HIV-infected MSM who engaged in non-injecting sexualized drug use, regardless of the presence of slamsex, the factors associated with more severe physical and psychopathological drug-related symptoms were smoked crystal-meth, GHB use (oral), ketamine, polydrug use, and self-reported depression and anxiety disorders. In particular, smoked crystal-meth was associated with higher rates of drug dependence and withdrawal symptoms. Moreover, participants who smoked crystal-meth more frequently had psychotic paranoid ideation and experienced loss of consciousness during or after drug use.

In addition to being injected intravenously, crystal-meth has been smoked by MSM at sex parties during the last decade, mainly in the London gay scene [22]. The potent disinhibiting effect of this drug has been related to high-risk sexual behaviors and an increase in the frequency of STIs, particularly HIV infection [23]. Furthermore, drug dependence has been described in MSM who inject crystal-meth and who are also more prone to comorbid psychiatric disorders and suicidal behavior [23]. Psychotic symptoms have been reported in other populations [24], although additional psychopathological symptoms induced by smoked crystal-meth in chemsex are uncommon.

Loss of consciousness was also associated with GHB and ketamine use in our sample. In addition, participants who used more than three drugs (polydrug use) had higher rates of loss of consciousness and paranoid ideation and tended to experience more pronounced symptoms of drug dependence. This observation must be taken into account, because GHB is usually consumed in combination with other drugs. GHB is frequently related to loss of consciousness, owing to its depressive effect on the central nervous system and the fact that it accumulates over time [2]. In addition, the combination of GHB with mephedrone, crystal-meth, and alcohol increases the risk of drug-drug interactions and overdose, with loss of consciousness and respiratory depression [2]. Although ketamine is a dissociative anesthetic that acts as a stimulant at low doses, with higher doses, polydrug use, and intravenous injection, it can increase the risk of loss of consciousness and cardiovascular toxicity in recreational settings [25] such as chemsex, as reported in the present study. Our results are congruent with the previously known effects of these drugs (crystal-meth, GHB, mephedrone). The results of the present study can help us to understand the role of each type of drug and of the route of administration in the severe psychopathological symptoms that may be experienced by people who engage in non-injecting sexualized drug use.

Finally, self-reported current diagnosed psychiatric disorders may have played a significant role among the participants who engaged in slamsex and in non-injecting sexualized drug use in our sample. Regardless of the presence of slamsex, those participants who self-reported current depression more frequently experienced withdrawal symptoms and suicidal ideation during or after drug use. Participants with current anxiety disorders also reported higher frequencies of withdrawal symptoms, suicidal ideation, and paranoid ideation in this context. Moreover, participants who engaged in slamsex were more likely to have been diagnosed with an anxiety disorder or a depressive disorder.

While there is evidence that HIV-positive MSM frequently present mental disorders such as depression, anxiety, and suicidal behavior, as well as drug-related disorders, there is little research on the effect of these variables on the health consequences of chemsex in this population. The initial published data suggest that HIV-positive MSM who engage in chemsex had a higher frequency of depression and anxiety disorders than HIV-positive MSM who did not [26]. Other studies on chemsex did not report psychopathological diagnoses, but rather analyzed the emotional distress and psychological discomfort associated with chemsex. It has been suggested that the vulnerability factors related to problematic use of drugs in chemsex may include the so-called minority stressors, such as negative internalized homophobia, fear of disapproval, experience of discrimination, and a negative self-concept [27].

Therefore, according to the syndemic approach, mental health disorders in HIV-infected MSM appear to increase vulnerability to drug abuse disorders and sexual risk behaviors in a framework by which disease outcomes and the social conditions that contribute to their proliferation sustain each other [27]. Consequently, a multidisciplinary approach is necessary to address the situation appropriately. Although the design of the present study and its limitations do not allow us to establish causality, in our opinion, the presence of depression and anxiety among HIV-positive MSM who engage in slamsex could act as a vulnerability factor that modulates the severity of physical and psychopathological symptoms during or after intravenous sexualized drug use. Moreover, people with previous mental health disorders may be more likely to start chemsex and become involved in high-risk practices such as slamsex for various reasons, such as low self-esteem, the need to be accepted by a group, self-neglect, and the desire to avoid negative feelings. These issues may be considered syndemic factors and should be further addressed in studies that delve deeper into mental health problems and enable us to evaluate possible mental disorders and their relationship with drug-use disorders [27]. Although we found that suicidal behaviors in participants who engaged in slamsex were associated with current depression and anxiety, they were also present in participants who reported anxiety and depression and engaged in non-injecting sexualized drug use. These findings suggest that people with depression or anxiety who engage in sexualized drug use are more vulnerable to developing suicidal behavior. In addition, intravenous use of specific drugs such as synthetic cathinones and other stimulants can trigger suicidal ideation in vulnerable individuals, mainly those who have already been diagnosed with depression or anxiety. Furthermore, as mentioned earlier, persons with current depression or anxiety may be more likely to use drugs intravenously, as they are less concerned about their personal welfare and the potential risk behaviors related to drug use. These associations suggest that the combination of psychopathology and intravenous drug use may facilitate suicidal ideation, suicide attempts, and psychotic symptoms in affected persons. However, while people with psychiatric disorders may be more vulnerable to suicidal ideation, intravenous consumption of drugs (such as mephedrone) and smoked crystal-meth can induce psychiatric symptoms as a result of their own effects. More research is needed to determine the relationship between these variables in people who engage in slamsex and non-injecting sexualized drug use.

We think it is important to evaluate the mental health of HIV-positive MSM alongside other routine evaluations conducted in HIV clinics. The detection of psychiatric disorders and their appropriate treatment can prevent potential mental and physical consequences of drug use in this population. In addition, approaches such as reducing the harm caused by drug use can be more effective in people who are not willing to stop sex-related drug use. It is necessary to create multidisciplinary approaches aimed at preventing and treating the potential consequences of slamsex and non-injecting sexualized drug use in this population.

To our knowledge, this is the first detailed analysis of drug-related and severe psychopathological symptoms in people who engage in slamsex. We also report data that increase our knowledge of the role of different types of drugs, routes of consumption, and psychiatric backgrounds, as well as drug-related symptoms and psychopathological symptoms associated with slamsex and non-injecting sexualized drug use among HIV-positive MSM.

Our study is subject to the limitations inherent to cross-sectional survey-based studies, especially response bias. Although we used limited time periods in questions that depended on memory, recall bias could distort the accuracy of the results. Furthermore, we cannot suggest causality because of the cross-sectional nature of the study. Longitudinal studies should be performed to compare our results in order to be able to discuss whether slamsex is related to previous psychopathology and contributes to the presence of drug-related and severe psychopathological symptoms in HIV-positive MSM. Another limitation is that the psychiatric diagnosis and drug-related symptoms were self-reported. Although the questionnaire specified previous or current psychiatric disorders diagnosed by a psychiatrist or other mental health specialist, the survey did not have standardized diagnostic scales. The exploratory nature of this study led to the inclusion of a large number of variables and the ad hoc design of the survey. In addition, the language used for questioning was not always standardized and had to be adjusted for the individual patient, with frequent use of slang terms. However, questions about substance dependence and withdrawal were elaborated following DSM-IV-Rev criteria. Future studies should include standardized screening scales for mental disorders, substance use disorders, presence of craving, and specific psychopathological symptoms to enable detailed measurement of specific variables.

Our results suggest that slamsex is relatively common, although it does not appear to be generalized among HIV-positive MSM who engage in chemsex in Spain. People who engage in slamsex more frequently seem to have high-risk practices associated with both drug use and sexual behaviors than people who engage in non-injecting sexualized drug use. Furthermore, people who engage in slamsex are more likely to experience drug-related psychopathological symptoms and symptoms of drug dependence. Moreover, the non-injected use of other substances, such as crystal-meth, GHB/GBL, and ketamine, and the presence of psychiatric disorders might also be related to severe consequences for the physical and mental health of HIV-positive MSM who engage in chemsex.
